# 3D-Printable and open-source modular smartphone visible spectrophotometer

**DOI:** 10.1016/j.ohx.2021.e00232

**Published:** 2021-09-21

**Authors:** Brandon J. Winters, Nick Banfield, Cassandra Dixon, Anna Swensen, Dakota Holman, Braxton Fillbrown

**Affiliations:** Bethel University, St. Paul, MN United States

**Keywords:** CAD, Computer Aided Design, LED, Light emitting diode, IoT, Internet of Things, 3D printing, Open-source, Spectroscopy, Internet of things (IoT), Computer aided design (CAD), Optics, Photonics, Citizen science

## Abstract

The past four decades have brought significant and increasingly rapid changes to the world of instrument design, fabrication, and availability due to the emergence of 3D printing, open-source code and equipment, and low-cost electronics. These, along with other technological advances represent a nexus in time ripe for the wide-spread production and availability of low-cost sophisticated scientific equipment. To that end, the design of a 3D printable and open-source, modular spectrometer is described. This specific instrument is distinctly different from others that have been reported in recent years in that it was designed outside of the “black box” paradigm of nearly all other commercially available and open-source spectrometers. This feature along with its design to be produced on low-end consumer-grade 3D printers and with parts available at nearly any local hardware store allow this instrument to further erode current barriers to instrument access. Additionally, the use cases presented here demonstrate similar capabilities to commercially available equipment at a fraction of the cost.

## Graphical abstract

**Figure:**
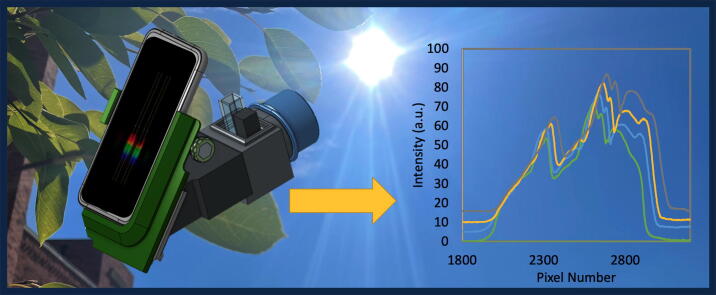

Specifications table:

**Table:** 

Hardware name	*Double-beam visible smartphone spectrophotometer*
Subject area	• Educational Tools and Open Source Alternatives to Existing Infrastructure
Hardware type	• Photonics Tools
Open Source License	This work is licensed under the Creative Commons Attribution-ShareAlike 4.0 International License. To view a copy of this license, visit http://creativecommons.org/licenses/by-sa/4.0/.
Cost of Hardware	Double-Beam Visible Spectrophotometer $25.53, Sources (Flashlight, LASER, penlights) $175.73, ELP USB Camera $64.26
Source File Repository	https://osf.io/7ewb3

## Introduction

It has been nearly 40 years since the invention of the first 3D printer and almost as long since the open-source movement began.[1], [2], [3] The simultaneous development of this technology and ideology may have been coincidental, but their individual importance in the development of vibrant communities adding to the body of knowledge surrounding myriad related markets can only be overshadowed by the mutually beneficial ways they rely on and add to one another. Indeed, it is increasingly true that a Makerspace, or similar facility, can be found in nearly every community and they almost always include at least one 3D printer and are operated with an ethos of open-source technology.[4], [5], [6] As these spaces, often public in accessibility, have been created and expanded, it has been accompanied by the development of cheap microprocessors for home-built electronics applications.[7] Today, rapid prototyping and manufacturing through quick design and fabrication utilizing free computer aided design (CAD) software and 3D printing paired with consumer interest in the internet of things (IoT) [8] and the widespread availability of programable microprocessors and breakout boards have resulted in an open-source climate where novice hobbyists are able to design, produce, and even commercialize sophisticated electronic devices. For the Analytical Chemist, this is an ideal atmosphere in which to produce laboratory equipment and instrumentation cheaper, more accessible and in some cases just as capable as what is available through instrument manufacturers. In fact, the globalization of markets, increased access and affordability in world-travel, and remarkable confluence of the aforementioned technologies presents an irresistible opportunity to develop cheap and portable instruments for chemical analysis that can be shared to communities of individuals who would not otherwise have such access due to factors such as geography, education, and economics, to name just a few.[9]

As a result, and building on the already well-developed world of open-source laboratory equipment,[9], [10], [11], [12], [13] a new modular, open-source, 3D printable, double beam spectrophotometer has been developed. The modularity of this instrument allows for rapid modification and adaptation to new applications along with providing better access to examine the fundamental underlying principles that allow such instruments to probe the chemical world. Additionally, the modular design provides an easy instrument interface for integration of various camera or detector technologies including smartphones, USB cameras, or point and shoot digital cameras, without requiring a full redesign of the instrument for rapid field changes. The open-source and 3D printable characteristics provide wide-spread access to the equipment, which has the potential to further augment current initiatives in community-based-science and break down barriers to scientific knowledge. The double-beam design reduces ambient noise and represents one of various state of the technology approaches to designing these instruments. The combination of each of these design considerations has resulted in an instrument that is cheap, easy to assemble and transport, reliable, and largely future-proof since it provides a starting point from which modest improvements can be rapidly made.

## Hardware description

The hardware reported here includes the necessary components to assemble a visible spectrophotometer that can utilize a wide range of cameras. For users intent on collecting data with only one type of camera, some of the following parts need not be printed (explanation of these considerations provided in assembly instructions). This hardware embodies the following properties:

• This hardware was designed using all Imperial units including those for purchased hardware, but metric dimensions in parenthesis are provided in mm.
• Many of the indicated purchased components were chosen for their widespread availability and simplicity, but cheaper alternatives are also available and have been used throughout development. These alternatives are not reported here since ease of use and implementation is a key goal of this work.
• This hardware is highly customizable as new or adapted components can be designed and integrated using CAD and entry-level 3D printers, the modular design aides in future modifications and adaptations as it potentially limits the number of parts requiring redesign. For example, the flashlight tube (DBS24) can be replaced with a simple redesign to adapt the 1.25 in. (31.75) o.d. of the Flashlight Adapter (DBS12) to a different flashlight diameter without requiring further modifications.
• The various camera mounts were designed to allow integration of at least the following iPhone models (SE, 6, 6 s, 7, 8, 8 s, and X), but other models may also fit the iPhone mount (DBS15) well. The Center Camera Phone Mount (DBS16) was designed generally to fit smartphones with cameras centered on their back face. For users with cameras or phones not supported by the available mounts, the Blank Mounting Plate (DBS25) is provided as a simple means to solvent weld a user-selected phone or camera case (potentially downloaded from online repositories like Tinkercad.com, Onshape.com, or instructables.com) to the spectrophotometer. Alternately, the original CAD files are available through Onshape.com for editing and modification.

## Design files

### Design files summary

**Table:** 

Design file name	File type	Open source license	Location of the file
DBS01 – Slit Tube	STL and STEP	Creative Commons Attribution-ShareAlike 4.0 International License	File downloads available at https://osf.io/7ewb3
DBS02 – Sample Holder	STL and STEP	Creative Commons Attribution-ShareAlike 4.0 International License	File downloads available at https://osf.io/7ewb3
DBS03 – Grating Mount	STL and STEP	Creative Commons Attribution-ShareAlike 4.0 International License	File downloads available at https://osf.io/7ewb3
DBS04 – Tube Adapter	STL and STEP	Creative Commons Attribution-ShareAlike 4.0 International License	File downloads available at https://osf.io/7ewb3
DBS05 – Source Adapter	STL and STEP	Creative Commons Attribution-ShareAlike 4.0 International License	File downloads available at https://osf.io/7ewb3
DBS06 - Block	STL and STEP	Creative Commons Attribution-ShareAlike 4.0 International License	File downloads available at https://osf.io/7ewb3
DBS07 – Cuvette Cover	STL and STEP	Creative Commons Attribution-ShareAlike 4.0 International License	File downloads available at https://osf.io/7ewb3
DBS08 – Fluorescence Cover	STL and STEP	Creative Commons Attribution-ShareAlike 4.0 International License	File downloads available at https://osf.io/7ewb3
DBS09 - Plug	STL and STEP	Creative Commons Attribution-ShareAlike 4.0 International License	File downloads available at https://osf.io/7ewb3
DBS10 – Side Cover	STL and STEP	Creative Commons Attribution-ShareAlike 4.0 International License	File downloads available at https://osf.io/7ewb3
DBS11 – LED Adapter	STL and STEP	Creative Commons Attribution-ShareAlike 4.0 International License	File downloads available at https://osf.io/7ewb3
DBS12 – Flashlight Adapter	STL and STEP	Creative Commons Attribution-ShareAlike 4.0 International License	File downloads available at https://osf.io/7ewb3
DBS13 – Flashlight Cradle	STL and STEP	Creative Commons Attribution-ShareAlike 4.0 International License	File downloads available at https://osf.io/7ewb3
DBS14 – LED Cradle	STL and STEP	Creative Commons Attribution-ShareAlike 4.0 International License	File downloads available at https://osf.io/7ewb3
DBS15 – iPhone Mount	STL and STEP	Creative Commons Attribution-ShareAlike 4.0 International License	File downloads available at https://osf.io/7ewb3
DBS16 – Center Camera Phone Mount	STL and STEP	Creative Commons Attribution-ShareAlike 4.0 International License	File downloads available at https://osf.io/7ewb3
DBS17 – USB Camera Mount	STL and STEP	Creative Commons Attribution-ShareAlike 4.0 International License	File downloads available at https://osf.io/7ewb3
DBS18 – Phone Clamp	STL and STEP	Creative Commons Attribution-ShareAlike 4.0 International License	File downloads available at https://osf.io/7ewb3
DBS19 – Knob	STL and STEP	Creative Commons Attribution-ShareAlike 4.0 International License	File downloads available at https://osf.io/7ewb3
DBS20 – USB Camera Base	STL and STEP	Creative Commons Attribution-ShareAlike 4.0 International License	File downloads available at https://osf.io/7ewb3
DSB21 – USB Camera Lid	STL and STEP	Creative Commons Attribution-ShareAlike 4.0 International License	File downloads available at https://osf.io/7ewb3
DSB22 – Slit Mount	STL and STEP	Creative Commons Attribution-ShareAlike 4.0 International License	File downloads available at https://osf.io/7ewb3
DSB23 – Slit Wedge	STL and STEP	Creative Commons Attribution-ShareAlike 4.0 International License	File downloads available at https://osf.io/7ewb3
DSB24 – Flashlight Tube	STL and STEP	Creative Commons Attribution-ShareAlike 4.0 International License	File downloads available at https://osf.io/7ewb3
DSB25 – Blank Mounting Plate	STL and STEP	Creative Commons Attribution-ShareAlike 4.0 International License	File downloads available at https://osf.io/7ewb3

Design File Descriptions:

**Table:** 

DBS01 – Slit Tube	STL and STEP files for part DBS01 – Slit Tube
DBS02 – Sample Holder	STL and STEP files for part DBS02 – Sample Holder
DBS03 – Grating Mount	STL and STEP files for part DBS03 – Grating Mount
DBS04 – Tube Adapter	STL and STEP files for part DBS04 – Tube Adapter
DBS05 – Source Adapter	STL and STEP files for part DBS05 – Source Adapter
DBS06 - Block	STL and STEP files for part DBS06 - Block
DBS07 – Cuvette Cover	STL and STEP files for part DBS07 – Cuvette Cover
DBS08 – Fluorescence Cover	STL and STEP files for part DBS08 – Fluorescence Cover
DBS09 - Plug	STL and STEP files for part DBS09 - Plug
DBS10 – Side Cover	STL and STEP files for part DBS10 – Side Cover
DBS11 – LED Adapter	STL and STEP files for part DBS11 – LED Adapter
DBS12 – Flashlight Adapter	STL and STEP files for part DBS12 – Flashlight Adapter
DBS13 – Flashlight Cradle	STL and STEP files for part DBS13 – Flashlight Cradle
DBS14 – LED Cradle	STL and STEP files for part DBS14 – LED Cradle
DBS15 – iPhone Mount	STL and STEP files for part DBS15 – iPhone Mount
DBS16 – Center Camera Phone Mount	STL and STEP files for part DBS16 – Center Camera Phone Mount
DBS17 – USB Camera Mount	STL and STEP files for part DBS17 – USB Camera Mount
DBS18 – Phone Clamp	STL and STEP files for part DBS18 – Phone Clamp
DBS19 – Knob	STL and STEP files for part DBS19 - Knob
DBS20 – USB Camera Base	STL and STEP files for part DBS20 – USB Camera Base
DSB21 – USB Camera Lid	STL and STEP files for part DBS21 – USB Camera Lid
DSB22 – Slit Mount	STL and STEP files for part DBS22 – Slit Mount
DSB23 – Slit Wedge	STL and STEP files for part DBS23 – Slit Wedge
DSB24 – Flashlight Tube	STL and STEP files for part DBS24 – Flashlight Tube
DSB25 – Blank Mounting Plate	STL and STEP files for part DBS25 – Blank Mounting Plate

## Bill of materials

Complete Bill of Materials available for download on Open Science Framework at https://osf.io/7ewb3

**Further details of materials and equipment used:**

▪ Onshape.com
  ▪ Free software used to design all printed pieces
▪ MakerBot Print
  ▪ Free software used to connect to the 3D printer as well as convert design files to printable files
▪ MakerBot 3D Replicator, 5th Gen.
  ▪ Entry-level 3D printer used for component production, prints exclusively PLA filament
▪ MakerBot Polylactic Acid Filament
  ▪ Filament used in 3D printer to print all pieces of the spectroscopy system

## Build instructions

**General hints and tips for assembly:**

• 3D printed components, especially those printed on entry-level printers, are subject to a significant amount of corner roughness or burrs. These, along with any roughness associated with the printed raft, should be removed using a razor blade prior to assembling any components.
• Specific component attribute sizes and their tolerances to fit with other parts are subject to change based on the type and model of 3D printer used. Test prints of several small pieces to confirm their fit are recommended.
• All parts were printed with 20% fill; it may be necessary to increase this parameter to increase structural rigidity depending on your particular printer and filament.
• While many printed pieces can be printed without supports, it may be necessary depending on the particulars of the printer in use to add supports, this should be decided on a case-by-case basis and limited information will be provided for this specific consideration.
• For some parts, their specific orientation on the printer bed had a significant impact on fit and function. This was largely a result of the horizontal grooves from each printed layer catching or locking on one another when they were intended to glide smoothly. Reprinting in a different orientation or smoothing these surfaces with a file or abrasive backed paper provided the intended fit.

**Assembling the slit mount sub-assembly:**

**Fig. 1:**
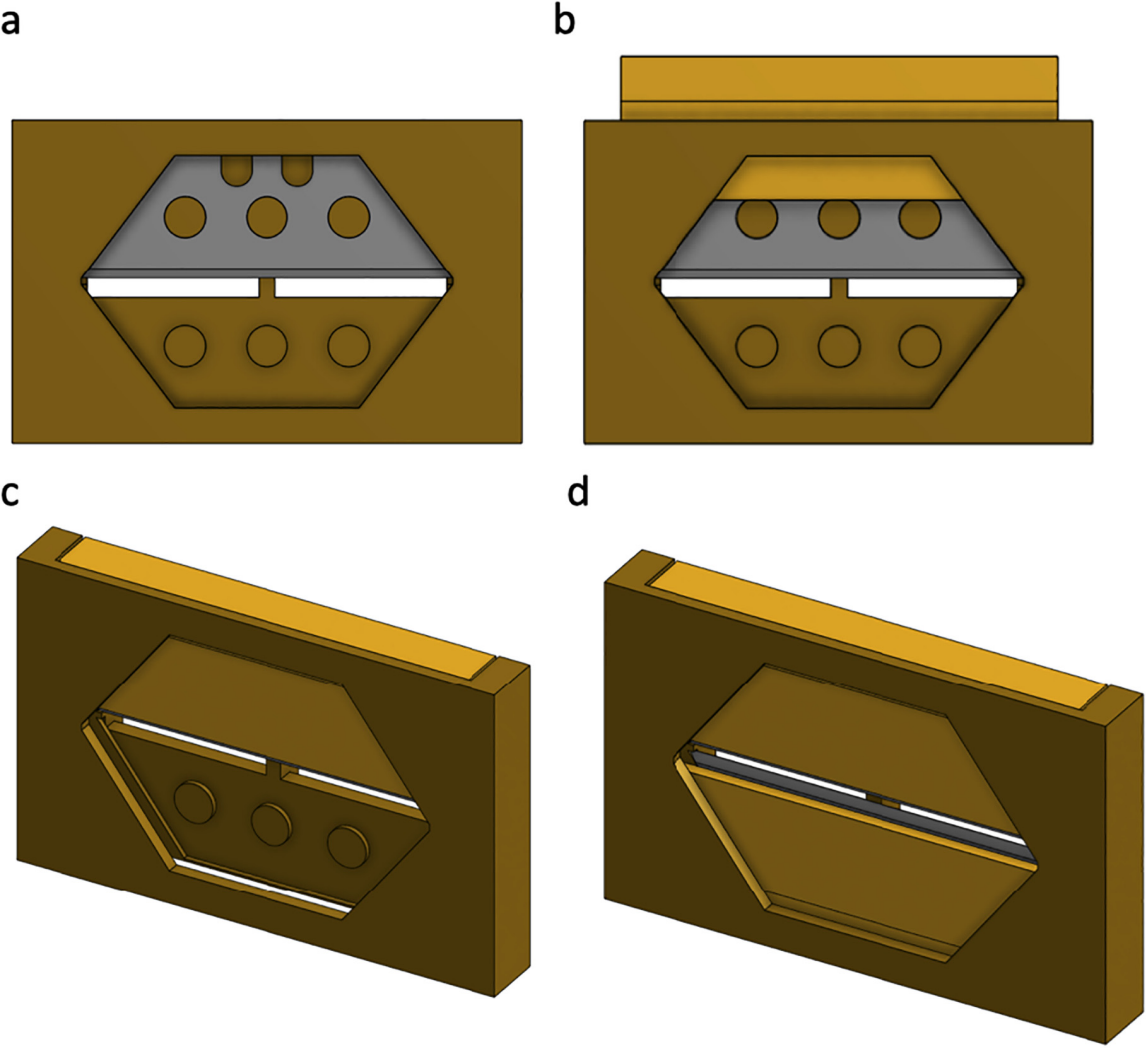
Illustration of the slit mount (DBS22) sub-assembly with (a) single blade (RS01) in position, (b) wedge (DBS23) partially inserted to secure blade, (c) wedge fully inserted, and (d) sub-assembly completed (2 × RS01 and DBS23).
iii. While holding the first blade firmly in place, slide one Slit Wedge (DBS23) into the associated slot of the Slit Mount (DBS22), Fig. 1b, and then tap the wedge fully into place with your fingers or a rubber mallet, Fig. 1c.
iv. Repeat steps ii. and iii. with the second blade so that your Slit Mount sub-assembly is completed as shown in Fig. 1d.

The slit mount requires assembly prior to placing it into the spectrophotometer. The following parts will be used in this sub-assembly: Slit Mount (DBS22), 2 × Slit Wedge (DBS23) and 2 × Miniature Utility Knife Blade (RS01)

i. The Miniature Utility Knife Blades (RS01) are very sharp and should be handled with care and caution.
ii. After ensuring the parts have printed correctly, are cleaned, and deburred, place one of the blades (RS01) into the Slit Mount (DBS22) such that the printed pegs of the mount align with the three holes of the blade, Fig. 1a.

**Assembling the smartphone mount sub-assembly:**

**Fig. 2:**
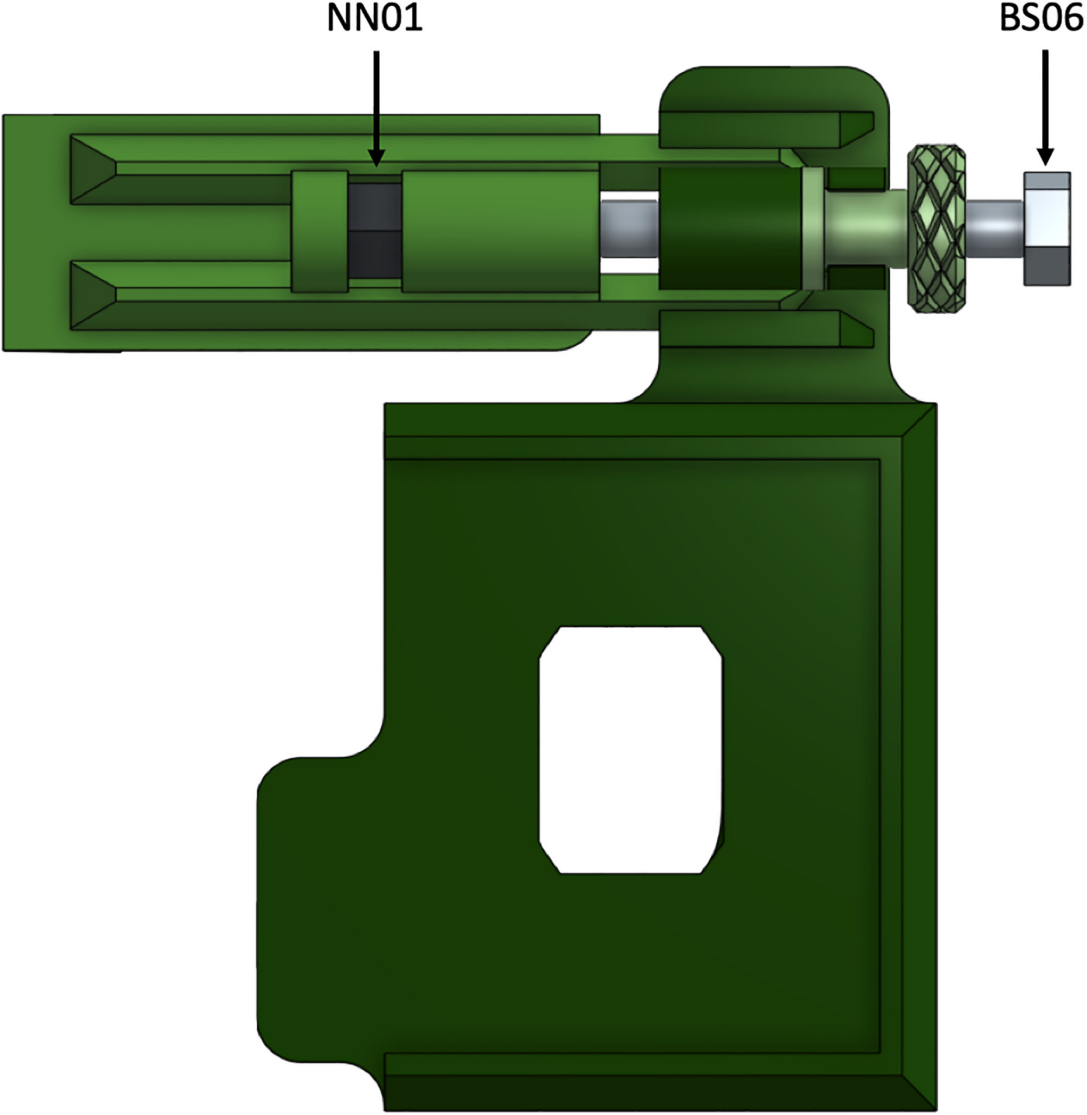
Illustration of the smartphone camera mount sub-assembly with steel bolt and nylon nut partially installed, includes DBS15, DBS18, and DBS19 as shown.
vii. Insert the Knob (DBS19) into the retaining clips of the Phone Mount and then slide the hex head bolt (BS06) through the knob and phone mount.
viii. With a nylon nut (NN01) held in the slot of the Phone Clamp, continue inserting the bolt until the threads of the bolt are in contact with the nut. The clamp position may need to by adjusted as you pair the head of the bolt into the knob and begin turning the bolt into the nut.
ix. Once this sub-assembly is complete, rotating the knob will affect the position of the clamp and will be used to secure the phone into the full spectrometer.

For any smartphone mount a camera mount sub-assembly is required. These instructions are identical for both types of mounts (DBS15 and DBS16). The following parts will be used in this sub-assembly: either iPhone Mount (DBS15) or Center Camera Phone Mount (DBS16), Phone Clamp (DBS18), one nylon ¼-20 hex nut (NN01), and one 3 in. ¼-20 hex head screw (BS06).

v. Prior to assembling the smartphone mount, the triangular slots and pegs of phone mount (DBS15 or DBS16) and Phone Clamp (DBS18) may require extra cleaning and fine-tuning. A tight fit that allows the clamp to move with little resistance in either direction can be achieved using a file or abrasive backed paper. After a tight fit has been achieved, a drop or two of a compatible oil can be added to ensure easy clamp motion.
vi. Slide the Phone Clamp (DBS18) into the Phone Mount (DBS15 or DBS16) as shown in Fig. 2.

**Assembling the spectrometer body (for iPhone):**

**Fig. 3:**
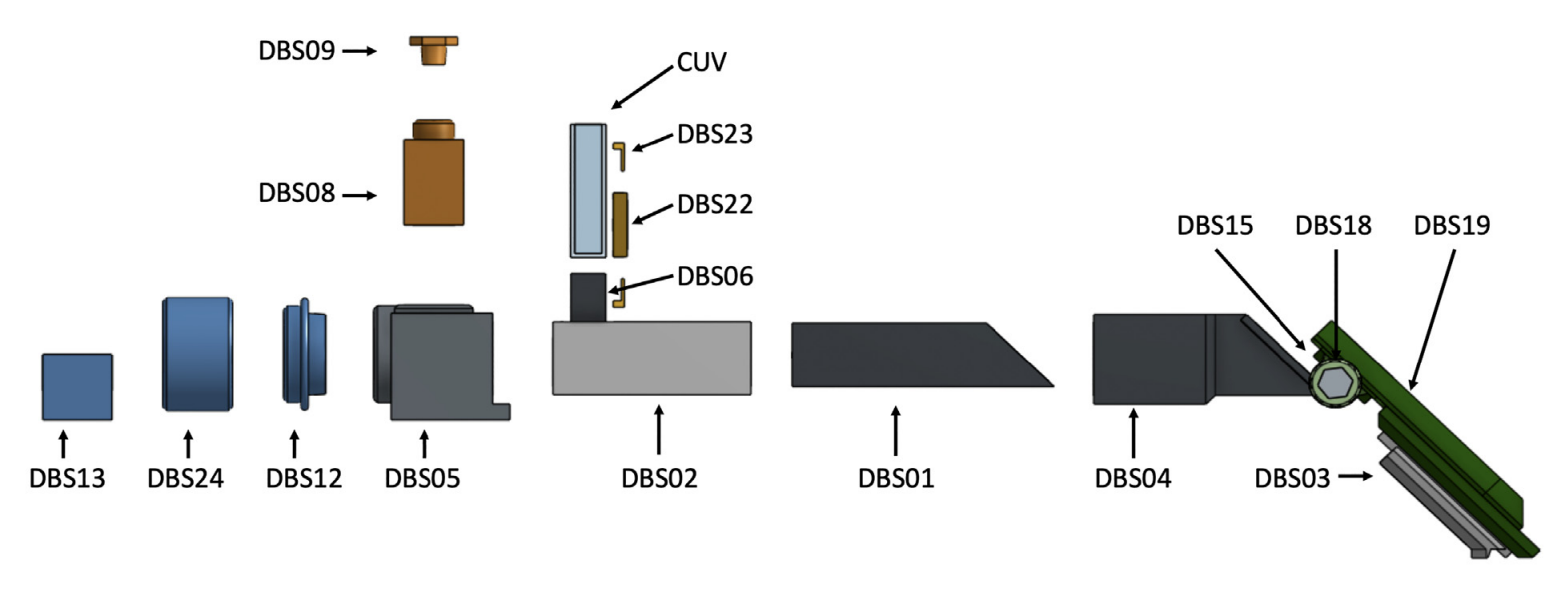
Blown-up view illustration of the full double-beam spectrophotometer for use with flashlight in absorbance mode, part designators indicated.

**Fig. 4:**
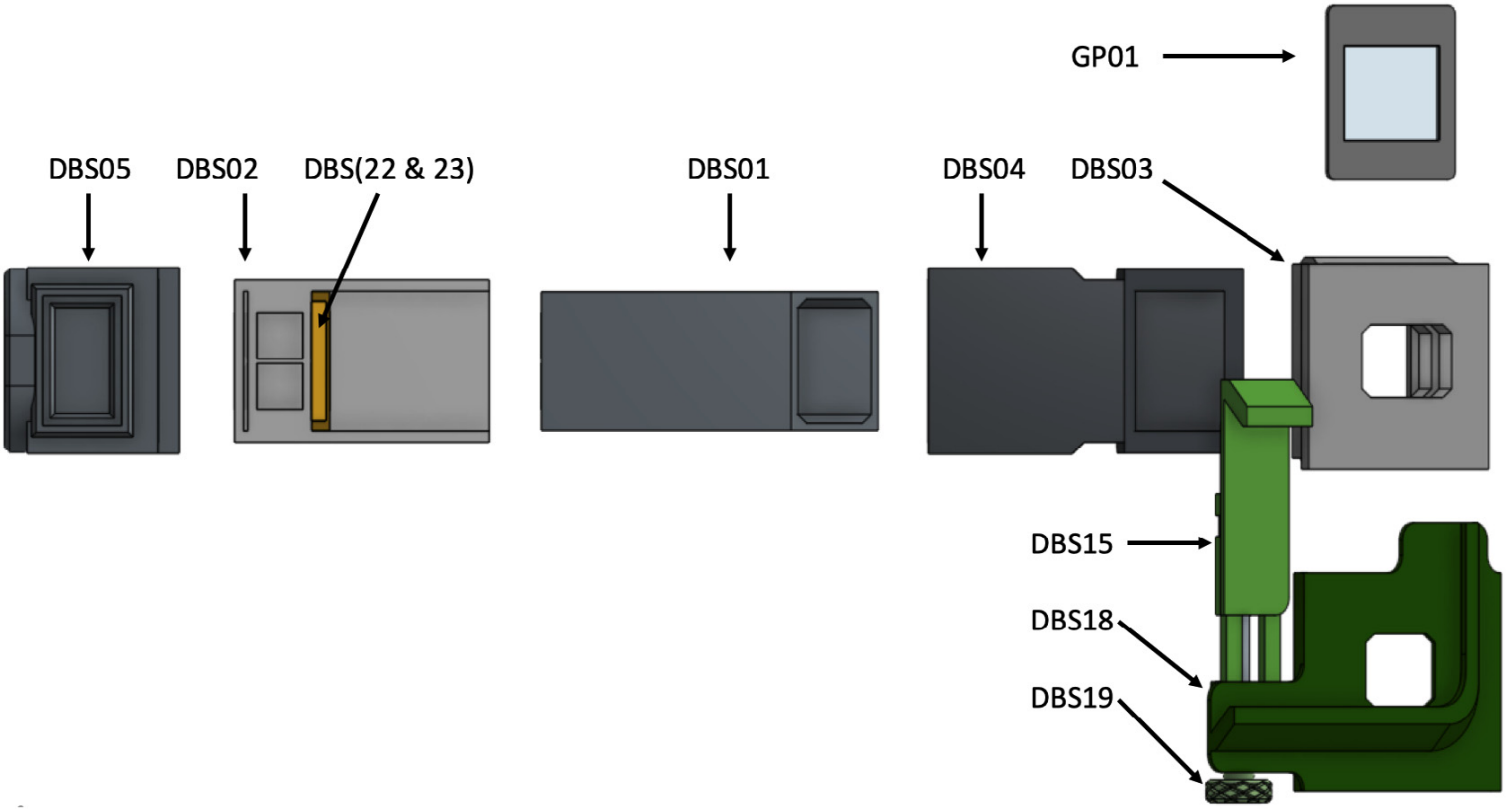
Blown-up view illustration of the core components of the double-beam spectrophotometer, part designators indicated.
xii. The Slit Tube (DBS01) is designed to interlock with the Grating Mount (DBS03) by extending past the end of the Tube Adapter (DBS04) when fully assembled as shown in Fig. 5. The Grating Mount should be slid onto the Tube Adapter (DBS04) and then the Tube Adapter with Grating Mount can be slid onto the already assembled Sample Holder with Slit Tube. These interlocking components provide better structural rigidity and help prevent ambient light from entering the apparatus.

For simplicity, Fig. 3 shows a blown-up illustration of the spectrophotometer setup for use with an iPhone camera. Each of the printed parts or sub-assemblies connects with the others via sliding friction fit, but since these parts were designed to overlap and in some cases interlock, they should be assembled and disassembled in the proper order.

x. Place the completed slit mount sub-assembly from step iv into the Sample Holder (DBS02) so that the sub-assembly is nearest the two sample slots.
xi. Slide the Slit Tube (DBS01) into the Sample Holder (DBS02) in the orientation shown below in Fig. 4.

**Fig. 5:**
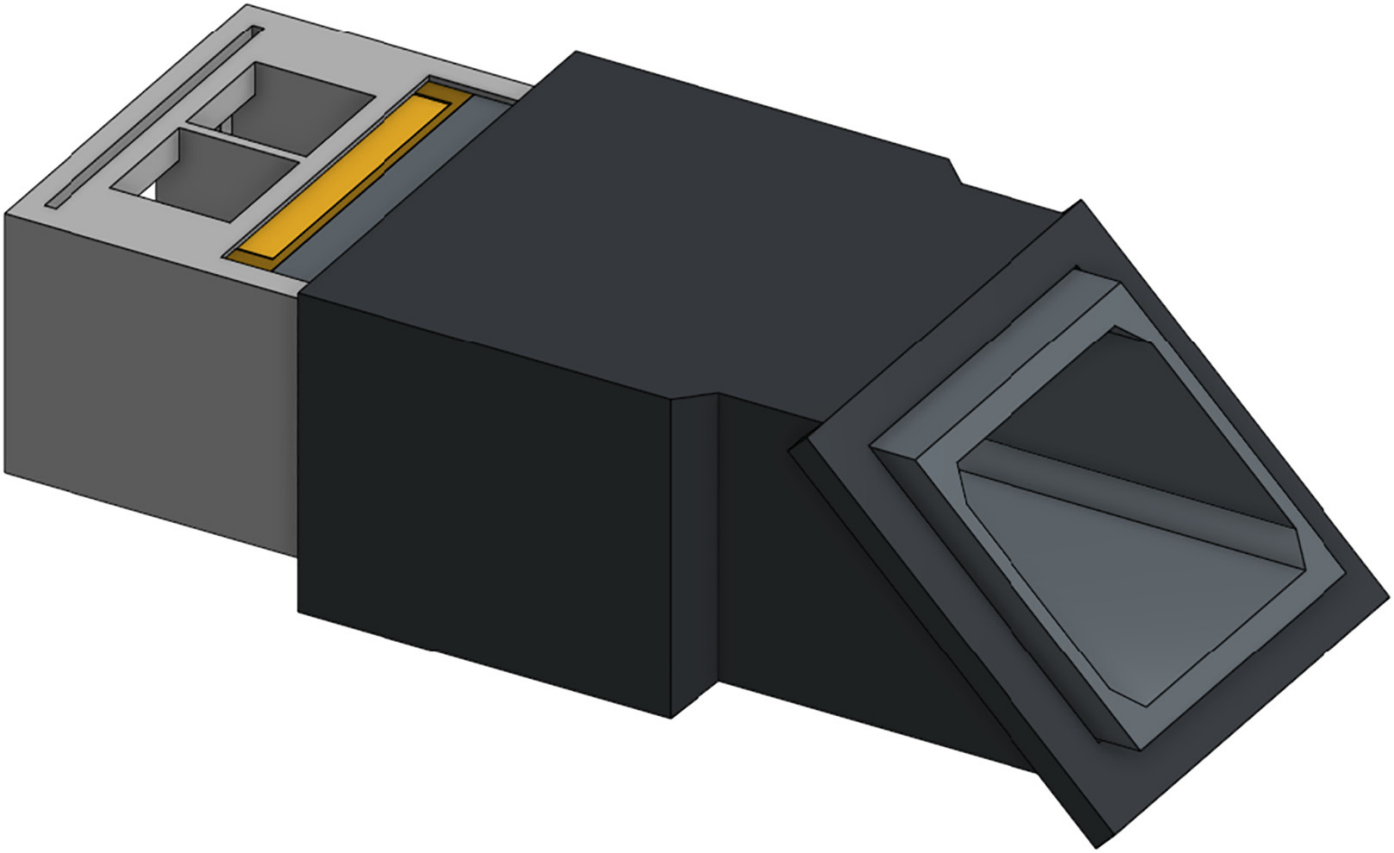
Illustration of the proper fit of sample holder (DBS02), slit mount (DBS22 and DBS23), slit tube (DBS01), and tube adapter (DBS04).
xiii. Slide the completed phone mount sub-assembly from step ix fully onto the Grating Mount (DBS03) and slide the Grating (GP01) into the grating slot in the side of the Grating Mount so that the blazes of the grating are horizontal.
xiv. For other detector/camera options simply replace the phone mount with an alternative camera mount such as the USB Camera Mounting Plate (DBS17) or alternately a modified Blank Mounting Plate (DBS25)
xv. Slide the Source Adapter (DBS05) over the sample slot end of the Sample Holder (DBS02) and press the assembly together from both ends to ensure all parts are seated correctly.
xvi. The apparatus, as assembled to this point, includes the core essential components (Fig. 6a). Further modifications using the modular components for Flashlight sources (DBS12, DBS13, and DBS24) (Fig. 6b), LED penlight sources (DBS11, DBS13, DBS14) (Fig. 6c), absorbance (DBS07) or fluorescence (DBS08 and DBS09) (Fig. 6d) measurements can be made using the remaining available printed parts.

**Fig. 6:**
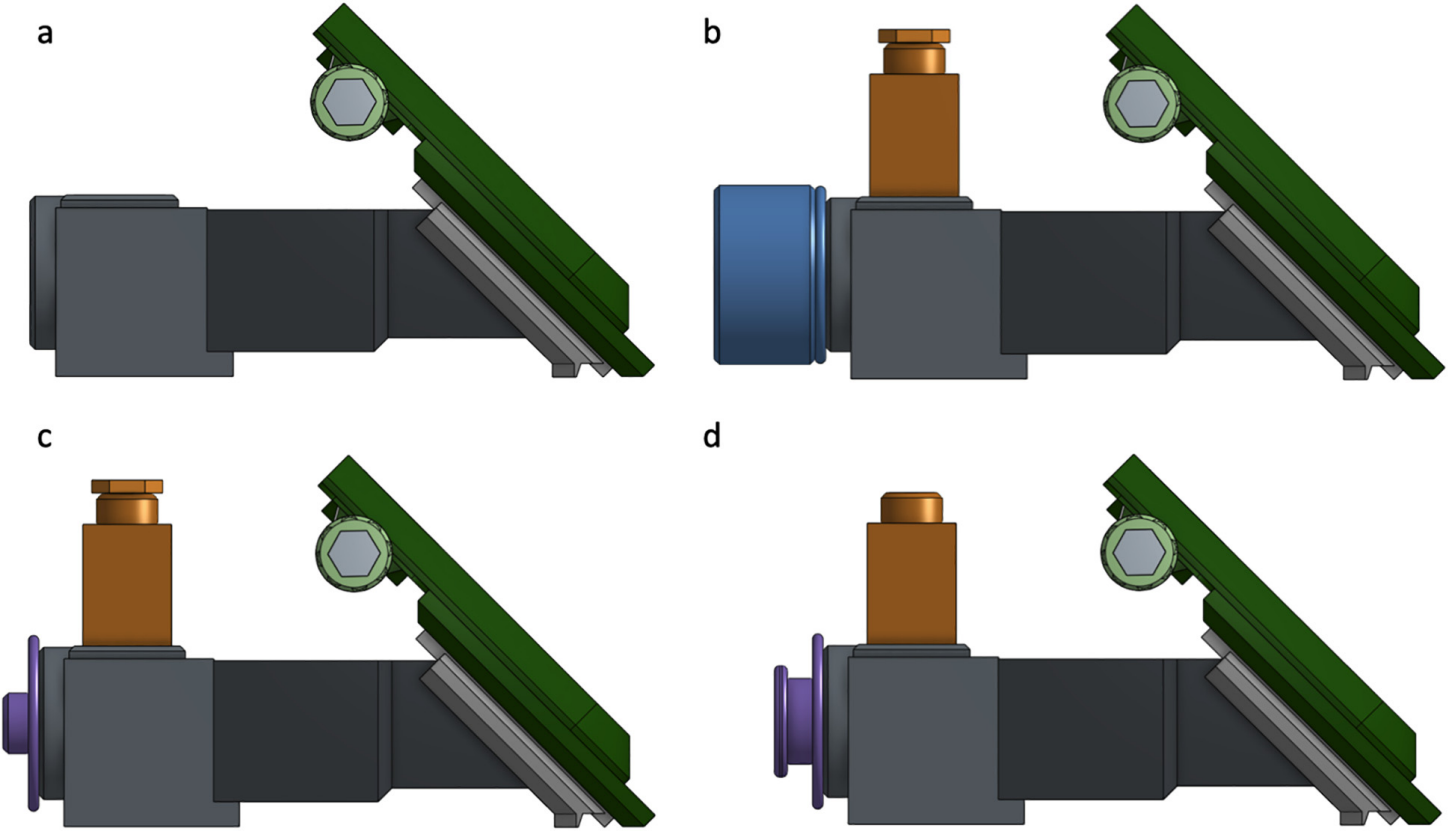
Illustration of the assembled DBS in various available configurations: (a) with essential core components, (b) for use with LED flashlight source, (c) for use with LED penlight source, (d) with source cap for fluorescence source above sample.

**Assembling the USB camera sub-assembly:**

The USB camera sub-assembly is designed for applications where a computer can be transported and used with the spectrophotometer. For many applications, as will be described further in validation methods, it is either not possible or undesirable to have the camera connected directly to a computer. The smartphone attachments are intended for these applications and those where a bring-your-own-device approach is desired. For this sub-assembly, the following parts will be utilized: USB Camera Base (DBS20), USB Camera Lid (DBS21), 4 × 18–8 steel sheet metal screws (SS01), ¼-20 nylon hex nut (NN01), and ELP USB Camera (USB01). For use with the spectrophotometer, this sub-assembly will also require the USB Camera Mounting Plate (DBS17)

xvii. After cleaning the printed parts, a single ¼-20 nylon nut (NN01) can be fitted into the sideway of the USB Camera Base (DBS20). This nut will not be used directly in the spectrophotometer, but allows the camera to be mounted to other optical components.
xviii. Follow the camera instructions to connect the USB power and data cable.
xix. The USB Camera (USB01) should friction fit into the USB Camera Base (DBS20) with the lens pointing down and out of the roughly ½ inch (12.4 mm) hole with chamfered conical opening.
xx. Align the USB cable so that it sits in the provided strain relief channel. Remove and rotate the USB camera to ensure there is little or no strain on its connection to the installed USB cable.
xxi. With the USB cable sitting in the strain relief channel, align the USB Camera Lid (DBS21) based on the orientation of the strain relief channel. Press it down into place and hold together by hand or with masking tape.
xxii. Using a small Philips screw driver, and the four 18–8 sheet metal screws (SS01) fasten the lid (DBS21) to the base (DBS20). For some printers it can be advantageous to shorten the screws using a wire or side cutter. In other cases, a small drop of solvent or fast drying glue can be used on the four corners of the lid to permanently close the camera case.
xxiii. Connect the USB camera to a computer and open the camera software. Turn the camera lens to adjust focus. This may need to be adjusted for the image on the spectrophotometer also, but can easily be as needed and should be verified periodically.

## Validation and characterization

### Initial lab validation

For initial validation of the spectrophotometer, two different tests were performed. First, calibration of the collected spectra with multiple laser and LED sources to determine the wavelength of an additional source. Second, the determination of an unknown solution concentration using a standard Beer’s Law plot.

For all analysis, the free software, ImageJ, was used to convert the collected image of the dispersed light into a plot of intensity vs. pixel by selecting the region of the image of interest and using the “plot profile” command. When peak maxima were analyzed irrespective of their corresponding wavelengths, the data was directly exported to processing software such as Microsoft Excel and used without further calibration. In cases where calibrated wavelength information was needed, a two-point calibration of the abscissa was performed using two LED lights, two laser pointers, or two lines from a fluorescent bulb. Functionally, each of these types of calibration sources are identical, but it is worth noting that although the phone mount for the DBS is designed to realign the phone in the same position each time the instrument is used, small differences are likely to exist. So, when precise results are desired, all measurements should be made in sequence without removing or realigning the phone/camera.

#### Instrument wavelength calibration

Once raw plot data for two calibration wavelengths was exported and plotted in Excel using the “data” and “copy all data” buttons of the plot profile, a calibration function was generated by comparing the pixel position of both wavelengths and producing a straight-line fit with the two ordered pairs: (p_1_,λ_1_) and (p_2_,λ_2_). Where p_1_ and p_2_ are the pixel location of the two calibration sources and λ_1_ and λ_2_ are their respective known wavelengths. Then the calibration line is:

(1) $$\lambda_{x}=\frac{\lambda_{2}-\lambda_{1}}{p_{2}-p_{1}}p_{x}+\lambda_{0}$$

where p_x_ is the pixel number from the camera image, λ_x_ is the calibrated wavelength, and λ_0_ is the y-axis intercept, which is solved for numerically using:

(2) $$\lambda_{0}=\lambda_{1}-p_{1}\left(\frac{\lambda_{2}-\lambda_{1}}{p_{2}-p_{1}}\right)$$

**Fig. 7:**
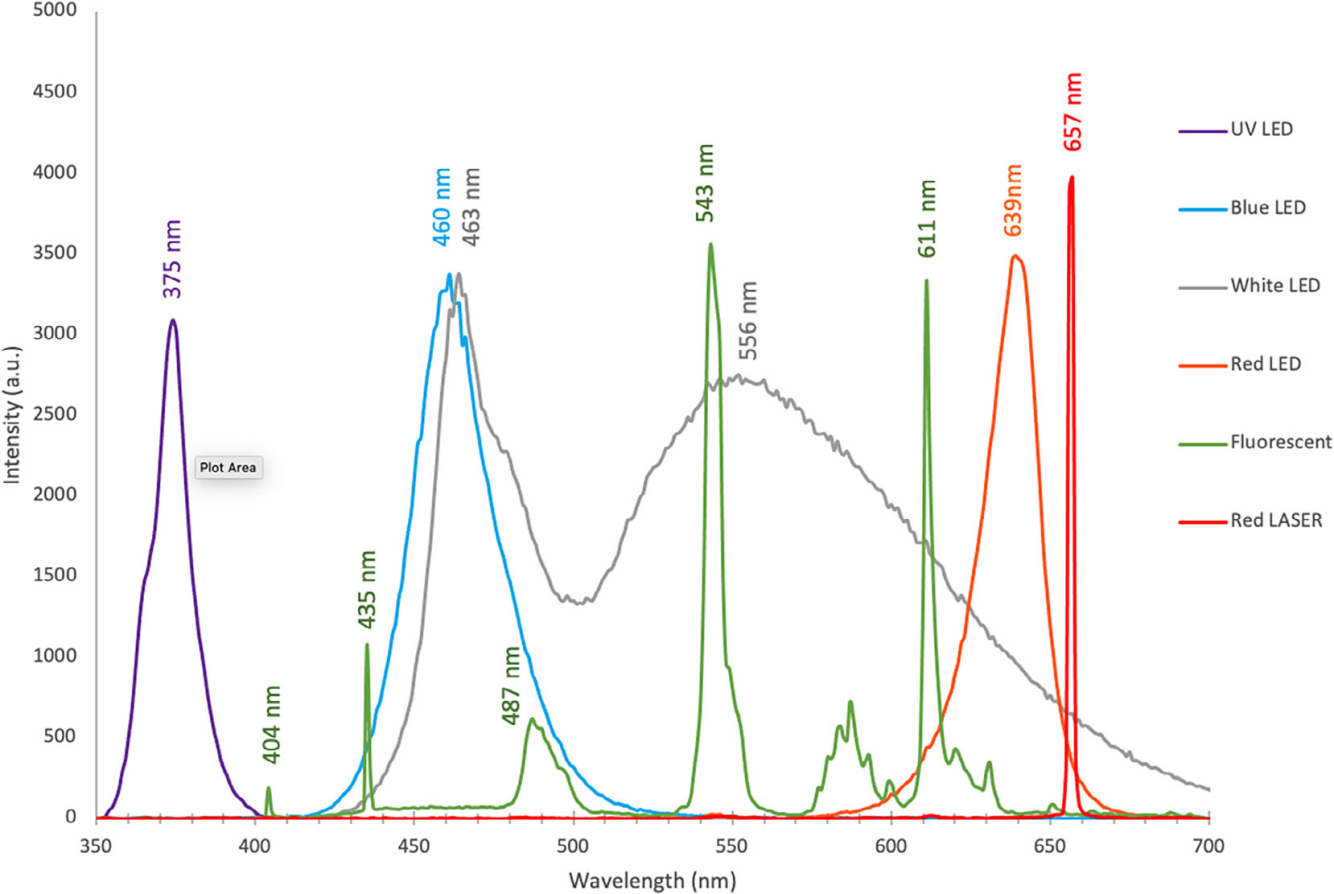
Reference spectra produced for field calibration of the DBS with various indicated light sources and their peak wavelengths.

The resultant calibration equation can be used in any data processing software to convert pixel number extracted from the raw images into calibrated wavelength. In preparation of field-use and analysis, a reference plot with spectra of portable sources and their known wavelengths was generated (Fig. 7).

#### Beer’s Law analysis

For analysis of an unknown solution concentration and validation of the DBS capability to quantify chemical concentrations, a 1.2 M stock solution of copper sulfate pentahydrate (CuSO_4_·5H_2_O, FM 249.69) was prepared by dissolving 30.0806 g of the solid (Sigma Aldrich, reagent grade) in a 100 mL class A, volumetric flask. A series of two-fold dilutions were then prepared, providing a total of 11 calibration solutions (Table 1) including the original 1.2 M stock.

**Table 1:** Calculated solution concentrations for Beer’s Law standard curve.

Sample ID	Conc (M)
stock solution	1.20472
1	0.60236
2	0.30118
3	0.15059
4	0.07529
5	0.03765
6	0.01882
7	0.00941
8	0.00471
9	0.00235
10	0.00118

**Fig. 8:**
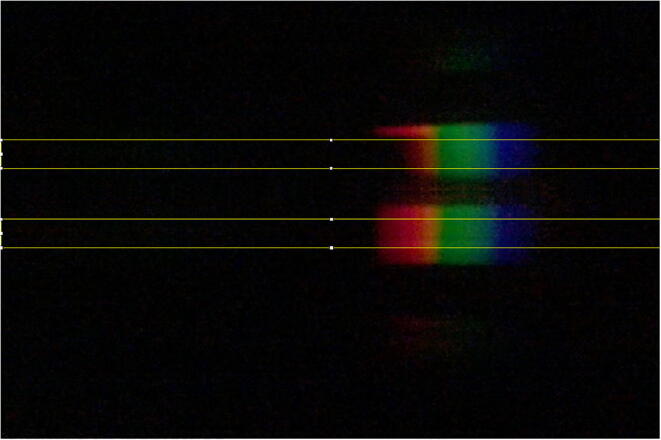
Smartphone image collected with the DBS, pixel regions for calculation of sample and reference spectra indicated.

**Fig. 9:**
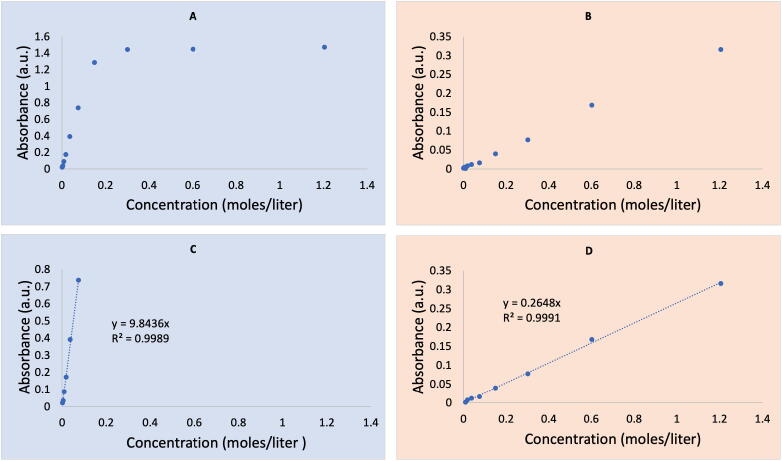
Plotted absorbance vs. concentration data for a series of copper sulfate dilutions from 1.2 to 1.2 × 10^-3^ M using (A) commercially available UV–Vis, (B) DBS, and background subtracted and isolated to only those points within the dynamic range for the commercially available spectrometer (C) and DBS (D.

Absorbance values for the 11 standard solutions were measured at 800 nm using the DBS with iPhone X and, for comparison and validation, a Perkin Elmer Lambda XLS UV–Vis spectrophotometer (PE Spec). The images acquired with the DBS were processed as described above by selecting a 200 pixel tall region across the full width of the images (yellow rectangles) for both the sample (top) and reference (bottom) as shown in Fig. 8. The profile plot data was exported for processing and the Absorbance was calculated as:

(3) $$\mathrm{Absorbance}=-\log_{10}\frac{P}{P_{0}}$$

where P is the peak intensity at 800 nm for the sample and P_0_ is the peak intensity at 800 nm for the reference sample. These absorbance values for the 11 standard solutions were then plotted for both instrumental techniques and background corrected so the line of best fit passed through the origin (Fig. 9). While not definitively determined, the upper limit of linearity for the PE Spec is clearly located around 0.2 M while the DBS does not appear to show an upper limit to the dynamic range. Additionally, both instruments show a likely limit of quantitation at the lowest concentrations (Fig. 9a). For these reasons, the data for each instrument was optimized to only utilize those datums that clearly fall within their respective linear dynamic range and least squares fit lines were fit accordingly (Fig. 9b). With this optimization, both techniques demonstrate remarkably similar results. Although the slope of the line for the PE Spec is roughly forty times that of the DBS making it much more sensitive, the full dynamic range of the DBS extends at least to 1.2 M copper sulfate, well beyond the 0.075 M upper limit of the PE Spec. There are clear trade-offs with these two instruments.

Based on the Beer’s Law plots for each instrument, the concentration of a single “unknown” was determined and calculated. The unknown was prepared by diluting 1.00 mL of stock solution with 10.00 mL of deionized water to bring the final total volume to 11.00 mL and produce a concentration of 0.10952 M. Absorbance for the PE Spec and DBS were measured to be 0.892 and 0.0239 respectively. Using the equations of best fit the PE Spec provided a concentration of 0.0906 M and the DBS provided 0.0903 M. While neither spectrophotometer provided a measured copper sulfate concentration that was particularly accurate, their proximity to each other suggests other influences on the final result and demonstrates the usefulness of the DBS as a possible replacement for teaching-grade UV–Vis spectrophotometers.

### Field validation

**Fig. 10:**
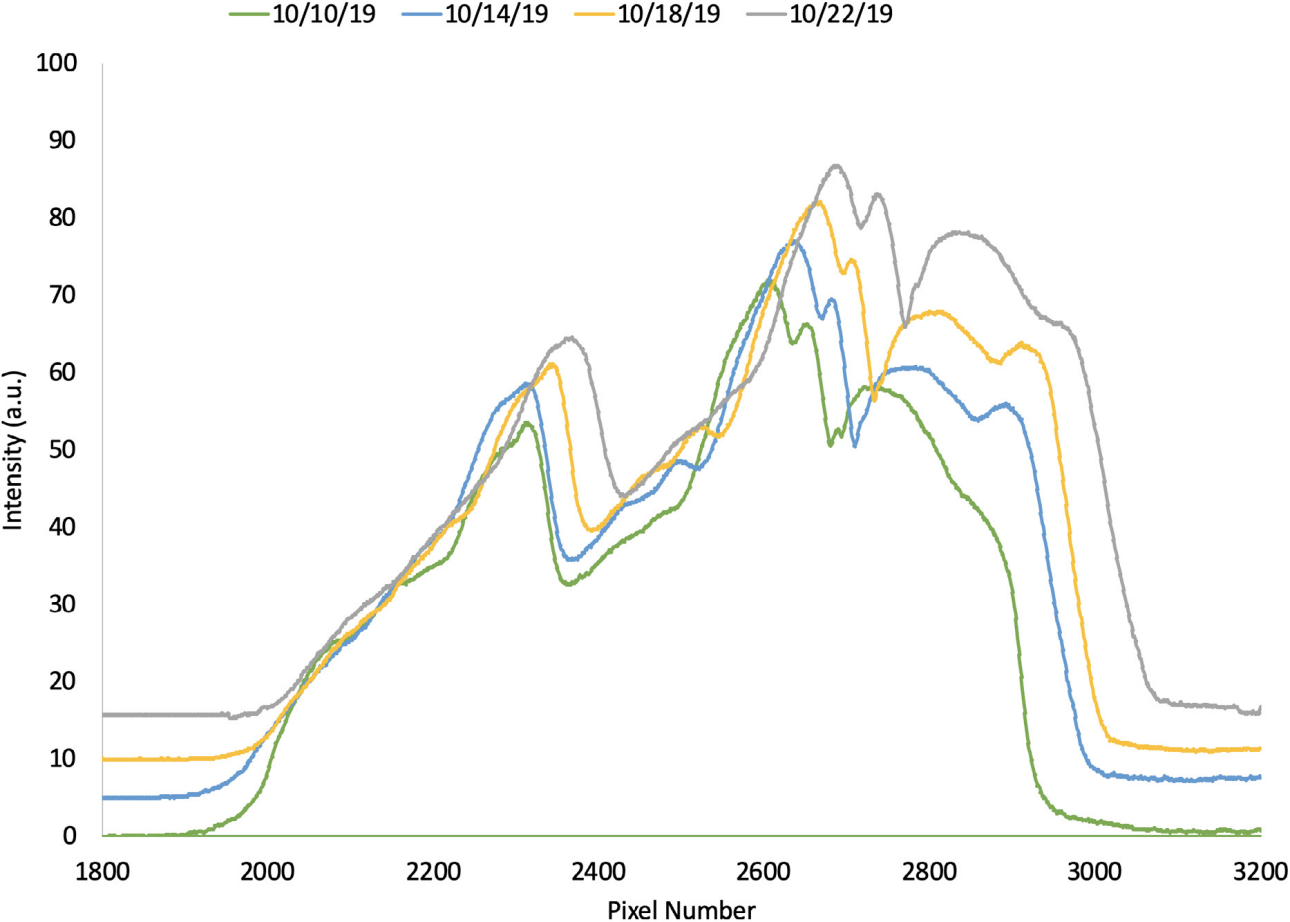
Uncalibrated spectra of the sun’s light collected on four different dates in the fall of 2019 with the DBS.

**Fig. 11:**
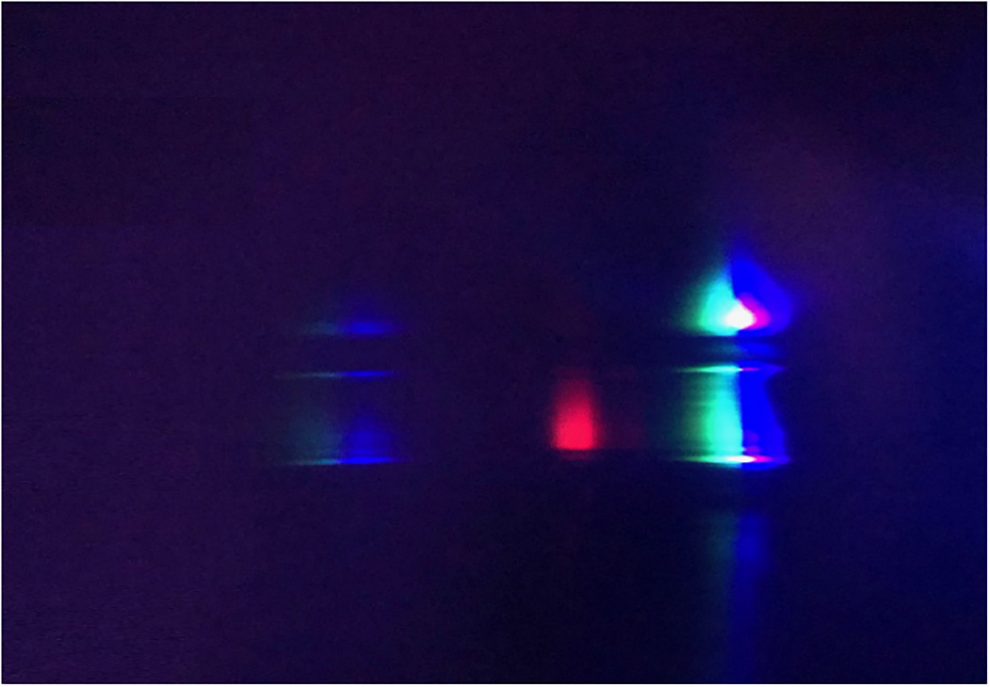
Image collected with a smartphone and the DBS of reference sample (top) and chlorophyll fluorescence (red signal on bottom) produced with alcohol extracts of leaves and a blue LED penlight. (For interpretation of the references to color in this figure legend, the reader is referred to the web version of this article.)

For field validation, the instrument has been used to observe the seasonal shift of the sun’s color spectrum (Fig. 10) and qualitatively observe the fluorescence of chlorophyll when irradiated with blue LED light (Fig. 11). While these results are not particularly quantitative in nature, they along with instrument calibration and quantitative determination of copper sulfate concentration represent much of the scope of functionality provided by the DBS.

## Conclusions

The DBS developed and demonstrated here represents a significant step in the arena of open-source instruments in that it has been designed from a modular framework that allows for rapid modification and adaptation while still producing an instrument with capabilities similar to those of other more expensive and commercially available ones. With commercially available visible spectrophotometers available for between $400 and $6000 (for teaching-grade units), this instrument brings the cost of ownership down significantly if the camera is assumed to already be purchased (in the case of a smartphone) or if a USB camera board is purchased and committed to the instrument. Since LEDs, laser pointers, and fluorescent light bulbs are so ubiquitous, it possible to argue their purchase cost should not be included in true cost to produce the DBS. Additionally, the adaptability for the DBS to utilize nearly any available camera whether it be a smartphone, USB webcam, or other device suggests the camera could be borrowed from another device and that cost should not be included either. In that case, the cost to produce a single DBS is roughly $25.

While there is still significant work that could be done to demonstrate yet further use cases and further validate the instrument’s performance characteristics, many of these will be specific to the work being done and cannot be fully explored here. The adaptability to utilize both smartphone and USB camera technologies along with both portable battery-operated and 120 V light sources further demonstrates the significant and robust flexibility of this instrument for use in both laboratory and field study situations. Further refinements or advanced to the DBS could include an adjustable-width slit apparatus, a self-contained camera and data processing unit for more streamlined analysis, geometric optimization of the source, slit, grating, and camera orientation to allow for a wider range of source powers, and inclusion of better and more expensive grating materials, to name just a few.

## Funding

This research did not receive any specific grant from funding agencies in the public, commercial, or not-for-profit sectors.

Dr. Brandon J. Winters earned his Masters and Doctoral degrees at the University of Minnesota where he specialized in the synthesis and characterization of aerosolized nanoparticles. After earning his Ph.D. in 2011 he accepted a faculty position at Bethel University in St. Paul, MN where he teaches General Chemistry, Quantitative Chemical Analysis, Instrumental Analysis, and a study-abroad course in Ecuador that addresses human impacts on the natural world. Brandon’s personal research interests are in the areas of materials science and analytical chemistry. Currently he is working on the development of a new thermal chemical synthetic route to grow and functionalize graphene oxide films for use in electronics and chemical sensors in addition to development of open-source and 3D printable laboratory instruments for both teaching and remote field use.

## Declaration of Competing Interest

The authors declare that they have no known competing financial interests or personal relationships that could have appeared to influence the work reported in this paper.
